# Adverse Safety Events in Emergency Medical Services Care of Children With Out-of-Hospital Cardiac Arrest

**DOI:** 10.1001/jamanetworkopen.2023.51535

**Published:** 2024-01-12

**Authors:** Carl O. Eriksson, Nathan Bahr, Garth Meckler, Matthew Hansen, Grace Walker-Stevenson, Ahamed Idris, Tom P. Aufderheide, Mohamud R. Daya, Ericka L. Fink, Jonathan Jui, Maureen Luetje, Christian Martin-Gill, Steven Mcgaughey, Jon Pelletier, Danny Thomas, Jeanne-Marie Guise

**Affiliations:** 1 Department of Pediatrics, Oregon Health and Science University, Portland; 2 Department of Emergency Medicine, Oregon Health and Science University, Portland; 3 Department of Pediatric Emergency Medicine, University of British Columbia, Vancouver, British Columbia, Canada; 4 Department of Pediatrics, University of British Columbia, Vancouver, British Columbia, Canada; 5 Public Health Division, Multnomah County Health Department, Portland, Oregon; 6 Department of Emergency Medicine, University of Texas Southwestern Medical Center, Dallas; 7 Department of Emergency Medicine, Medical College of Wisconsin, Milwaukee; 8 Department of Critical Care Medicine, University of Pittsburgh Medical Center, Pittsburgh, Pennsylvania; 9 Department of Emergency Medicine, University of Pittsburgh, Pittsburgh, Pennsylvania; 10 Department of Pediatrics, Akron’s Children’s Hospital, Akron, Ohio; 11 Department of Pediatrics, Medical College of Wisconsin, Milwaukee; 12 Department of Obstetrics, Gynecology, and Reproductive Biology, Beth Israel Deaconess Medical Center and Harvard Medical School, Boston, Massachusetts

## Abstract

**Importance:**

Survival for children with out-of-hospital cardiac arrest (OHCA) remains poor despite improvements in adult OHCA survival.

**Objective:**

To characterize the frequency of and factors associated with adverse safety events (ASEs) in pediatric OHCA.

**Design, Setting, and Participants:**

This population-based retrospective cohort study examined patient care reports from 51 emergency medical services (EMS) agencies in California, Georgia, Oregon, Pennsylvania, Texas, and Wisconsin for children younger than 18 years with an OHCA in which resuscitation was attempted by EMS personnel between 2013 and 2019. Medical record review was conducted from January 2019 to April 2022 and data analysis from October 2022 to February 2023.

**Main Outcomes and Measure:**

Severe ASEs during the patient encounter (eg, failure to give an indicated medication, 10-fold medication overdose).

**Results:**

A total of 1019 encounters of EMS-treated pediatric OHCA were evaluated; 465 patients (46%) were younger than 12 months. At least 1 severe ASE occurred in 610 patients (60%), and 310 patients (30%) had 2 or more. Neonates had the highest frequency of ASEs. The most common severe ASEs involved epinephrine administration (332 [30%]), vascular access (212 [19%]), and ventilation (160 [14%]). In multivariable logistic regression, the only factor associated with severe ASEs was young age. Neonates with birth-related and non–birth-related OHCA had greater odds of a severe ASE compared with adolescents (birth-related: odds ratio [OR], 7.0; 95% CI, 3.1-16.1; non–birth-related: OR, 3.4; 95% CI, 1.2-9.6).

**Conclusions and Relevance:**

In this large geographically diverse cohort of children with EMS-treated OHCA, 60% of all patients experienced at least 1 severe ASE. The odds of a severe ASE were higher for neonates than adolescents and even higher when the cardiac arrest was birth related. Given the national increase in out-of-hospital births and ongoing poor outcomes of OHCA in young children, these findings represent an urgent call to action to improve care delivery and training for this population.

## Key Points

**Question:**

What are the frequency of and factors associated with severe adverse safety events in emergency medical services care of children with out-of-hospital cardiac arrest?

**Findings:**

In this cohort study of 1019 encounters, 60% of patients experienced at least 1 severe adverse safety event. Neonates had increased odds of a severe adverse safety event compared with adolescents.

**Meaning:**

These findings suggest that decreasing severe adverse safety events may improve current poor outcomes for children with out-of-hospital cardiac arrest, especially younger children.

## Teaser

This cohort study used medical record review to characterize the frequency of and factors associated with adverse safety events in pediatric out-of-hospital cardiac arrest treated by emergency medical services (EMS).

## Introduction

Pediatric out-of-hospital cardiac arrest (OHCA) is a devastating problem that affects 15 000 to 25 000 children per year in the United States,^1,2,3^ with dismal hospital survival at 8%.^2^ Adult OHCA and pediatric in-hospital cardiac arrest survival have improved over the last 15 years,^4,5^ without improvement in pediatric OHCA survival.^2^ While the causes of OHCA in children are often different than in adults,^6^ quality of care and systems issues may contribute to this disparity in outcomes. One study found significant geographic variation in survival from pediatric OHCA after controlling for demographic and established prognostic factors,^2^ supporting the hypothesis that differences in quality of care could contribute to variability in outcomes.

Pediatric OHCA cases are rare for any individual emergency medical service (EMS) clinician and infrequent within EMS agencies. The low frequency, high-stakes nature of pediatric OHCA and the complex and time-dependent care required present significant challenges for EMS personnel. Previous studies have documented concerns regarding the quality and safety of care delivered for pediatric OHCA patients.^7,8^ These include timeliness and accuracy of medication administration, cardiopulmonary resuscitation (CPR) quality, and complications related to advanced airway management.^9,10^ However, prior research has generally been limited to single geographic locations and limited sample sizes. The objective of this study was to evaluate the quality of resuscitative care for pediatric OHCA among a large geographically diverse cohort of patients, characterizing prevalence, variation, and factors associated with adverse safety events (ASEs) in pediatric OHCA. Based on prior work, we hypothesized that severe ASEs would be prevalent in this high-risk population and that we could identify factors associated with their occurrence.

## Methods

### Study Design

This cohort study was a retrospective medical record review of pediatric OHCA EMS care episodes that occurred between 2013 and 2019. The institutional review board at Oregon Health & Science University granted a waiver for informed consent due to minimal risk. We have previously reported our methods for this study, including data collection, analysis, and sample size calculations.^11^ Medical record review was conducted from January 2019 to April 2022 and data analysis from October 2022 to February 2023. Reporting of this study conforms to the Strengthening the Reporting of Observational Studies in Epidemiology (STROBE) reporting guideline statement.^12^

### Study Sites and Participants

We collected data from EMS agencies from 7 US regions: Portland (Oregon), Pittsburgh (Pennsylvania), Milwaukee (Wisconsin), San Bernardino (California), Atlanta (Georgia), Oxnard (California), and Dallas (Texas). Sites were selected based on geographic diversity and availability of electronic patient care reports (PCRs) with the ability to screen cases using standardized criteria.

Inclusion criteria were (1) younger than 18 years and (2) OHCA. Exclusion criteria were (1) declared dead on EMS arrival or signs of rigor mortis or dependent lividity on EMS arrival, (2) resuscitation efforts stopped and death declared within 10 minutes of EMS arrival, (3) no CPR or defibrillation performed by EMS, or (4) incomplete record or insufficient documentation to review. The first 3 exclusion criteria were established to exclude nonviable patients where care was either not delivered or quickly stopped. Each included EMS care episode or encounter was considered 1 study encounter, regardless of the number of instances of cardiac arrest within that episode.

### Outcome Measurement

The primary outcome was the presence of a severe ASE (with the potential to cause permanent or severe harm) in 1 of 6 domains of care: assessment, clinical decision-making, nonairway procedures, airway procedures, medications, and fluids. We previously created a taxonomy of ASEs based on American Heart Association (AHA) resuscitation guidelines and expert consensus, including criteria for determining severity and preventability.^11^ Our pediatric prehospital safety event detection system (PEDS) medical record review tool was modified for OHCA^13^ and was used to identify ASEs and capture clinical and demographic data. The tool captures details of key interventions and includes basic decision support to help identify common ASEs.

### Medical Record Review

We obtained EMS PCRs from participating agencies and redacted identifying information before loading into an electronic database for review. Data were collected and managed at Oregon Health & Science University using REDCap electronic data capture tools. We followed the Utstein format^14^ for reporting OHCA characteristics and Complex Chronic Conditions (version 2)^15^ to characterize the presence of an underlying disease. Sustained return of spontaneous circulation (ROSC) was defined as ROSC lasting until the end of the EMS care episode. For patient care episodes with multiple PCRs, we matched PCRs based on date of OHCA and clinical details and analyzed multiple PCRs together.

Study team research assistants entered information from PCRs into the PEDS tool on key elements of care and their timing, relying on structured data elements (rather than narrative) when possible; questions regarding data clarity or inaccuracy were resolved by consensus of our team of core reviewers (M.H., G.M., and C.E.). We recruited clinical reviewers who were pediatric emergency medicine or critical care physicians. Reviewers were trained to use the PEDS tool and achieved 80% agreement (based on convention^16^ and our prior studies) on the presence or absence of severe ASEs in 10 PCRs that had been reviewed by our core reviewers. During the study period, we periodically provided additional training to reviewers when questions arose or when we identified opportunities to improve consistency. In addition, we provided reviewers with a matrix of criteria for common ASEs to aid determination and improve consistency.^11^ Reviewers did not review care episodes from their local EMS systems to avoid potential conflicts of interest.

We used Excel’s RAND() function to randomly assign all PCRs for single review and 8% for dual review. Dual review PCRs were reviewed by a clinical reviewer and by 1 of our core reviewers; disagreements were adjudicated by our 3 core reviewers and consensus among this group was considered the gold standard. We measured percentage agreement between reviewers in the presence of a severe ASE in each domain due to limitations in Cohen κ when expected agreement is high due to low or high prevalence.^16^

### Statistical Analysis

We used descriptive statistics to evaluate characteristics of patients, OHCA events, EMS treatment and outcomes, and the number of severe ASEs. We explored the association between the presence of a severe ASE (outcome) and characteristics of patients and OHCA events using a multilevel logistic regression model clustered by study site. The following covariates were selected a priori because they aligned with Utstein criteria,^14^ were clinically relevant, and were reliably reported with low missing values: patient age and sex, witnessed arrest, shockable arrest, receipt of bystander CPR, public location, presence of complex chronic conditions, time to scene, and time on scene. We did not include race and ethnicity because they were often missing. Variables were modeled as random effects because they could affect the association with ASEs differently based on site. Due to a large discrepancy between the number of PCRs from different agencies, we clustered by study site rather than agency to generate more comparable sample sizes. Data analysis was conducted with Stata version 15 (StataCorp).

We assessed missingness and found that all variables were under 5% missing, except for time to scene (5.5% missing) and time on scene (7.7% missing). After exploring different imputation strategies including setting missing values as the mean, minimum, and maximum value and finding no significant difference between odds ratios (ORs) generated using different imputation strategies, our final model excluded samples with missing values.

## Results

**Figure 1.**
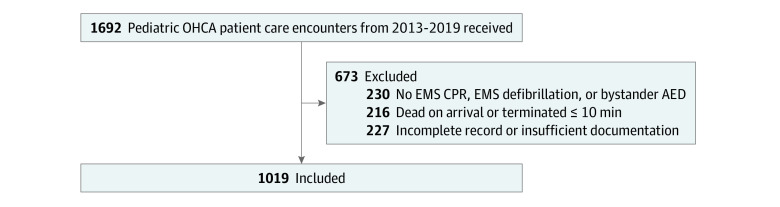
Flow Diagram of Pediatric Out-of-Hospital Cardiac Arrest (OHCA) Encounters AED indicates automated external defibrillator; CPR, cardiopulmonary resuscitation; EMS, emergency medical services.

We included 1019 encounters of EMS-treated pediatric OHCA from 51 EMS agencies from 2013 to 2019 (Figure 1). Overall, 42 patients (4%) had OHCA associated with birth outside of a hospital, and 465 patients (46%) were younger than 12 months (Table 1). EMS clinicians reported 293 patients (29%) were Black or African American, 231 (23%) were White, and race was not reported for 417 patients (41%). Ethnicity was unknown or not reported for 716 patients (70%). A total of 256 patients (25%) had a known complex chronic condition. Asystole was the most frequent first documented rhythm.

**Table 1.** Characteristics of 1019 Encounters for EMS-Treated Pediatric Out-of-Hospital Cardiac Arrests From 2013 to 2019

Characteristic	Encounters, No. (%)					
	Neonates		Infants (29 d-11 mo) (n = 380 [37%])	Children (1-11 y) (n = 354, [35%])	Adolescents (12-17 y) (n = 200, [20%])	Total (N = 1019)
	Births (n = 42 [4%])	Nonbirth (<28 d) (n = 43 [4%])				
Patient characteristics						
Age, median (IQR)	0	14 (9-23) d	3 (2-5) mo	3 (2-7) y	15 (14-17) y	1 (0-9) y
Sex						
Female	17 (40)	21 (49)	147 (39)	137 (39)	70 (35)	392 (38)
Male	25 (60)	22 (51)	233 (61)	217 (61)	130 (65)	627 (62)
Complex chronic condition	14 (33)	4 (9)	59 (16)	134 (38)	45 (23)	256 (25)
Cardiac arrest characteristics						
Witnessed arrest	34 (81)	9 (21)	70 (18)	148 (42)	77 (39)	338 (33)
Home location	36 (86)	35 (81)	318 (84)	265 (75)	133 (67)	787 (77)
Bystander CPR performed before EMS arrival	17 (40)	28 (65)	267 (70)	233 (66)	136 (68)	681 (67)
Bystander defibrillation performed	0	0	1 (<1)	2 (1)	10 (5)	13 (1)
Arrest after arrival of 911 responder	10 (24)	0	4 (1)	28 (8)	12 (6)	54 (5)
First documented rhythm						
Shockable (ventricular tachycardia/fibrillation)	0	0	14 (4)	17 (5)	29 (15)	60 (6)
Asystole	12 (29)	32 (74)	301 (79)	245 (69)	116 (58)	706 (69)
PEA	9 (21)	11 (26)	41 (11)	69 (19)	40 (20)	170 (17)
Bradycardia	7 (17)	0 (0)	4 (1)	6 (2)	1 (1)	18 (2)
Other or not documented	14 (33)	0 (0)	20 (5)	17 (5)	14 (7)	65 (6)
EMS response characteristics, median (IQR)						
Response time from activation to scene, min	6 (4-7)	5 (4-6)	5 (4-7)	5 (4-7)	5 (4-8)	5 (4-7)
Time on scene, min	11 (9-17)	14 (9-23)	12 (7-19)	13 (8-20)	18 (12-23)	14 (8-21)
Transport time from scene to hospital, min	10 (7-15)	10 (7-14)	10 (6-14)	10 (6-14)	10 (6-16)	10 (6-15)
EMS treatment characteristics						
Airway, bag, or mask	35 (83)	37 (86)	326 (86)	293 (83)	161 (81)	852 (84)
Intubation attempt	12 (29)	19 (44)	214 (56)	179 (51)	105 (53)	529 (52)
Supraglottic airway attempt	2 (5)	8 (19)	34 (9)	48 (14)	57 (29)	149 (15)
Defibrillation by EMS	1 (2)	3 (7)	19 (5)	32 (9)	43 (22)	98 (10)
Vascular access attempt	11 (26)	40 (93)	355 (93)	342 (97)	198 (99)	946 (93)
Vascular access success No. successes/ No. attempts (%)	6/11 (55)	33/40 (82)	317/355 (89)	312/342 (91)	186/198 (94)	854/946 (90)
IV attempt	3 (7)	7 (16)	52 (14)	100 (28)	114 (57)	276 (27)
IV success, No. successes/No. attempts (%)	0/3	3/7 (43)	21/52 (40)	55/100 (55)	86/114 (75)	165/276 (60)
IO attempt	10 (24)	39 (91)	341 (90)	295 (83)	129 (65)	814 (80)
IO success, No. successes/No. attempts (%)	6/10 (60)	32/39 (82)	300/341 (88)	271/295 (92)	120/129 (93)	729/814 (90)
Epinephrine given	7 (17)	34 (79)	321 (84)	286 (81)	168 (84)	816 (80)
Outcomes						
Sustained ROSC	12 (29)	6 (14)	40 (11)	74 (21)	70 (35)	202 (20)
Death declared before arrival to the hospital	5 (12)	13 (30)	77 (20)	40 (11)	34 (17)	169 (17)

Abbreviations: CPR, cardiopulmonary resuscitation; EMS, emergency medical services; IO, intraosseous; IV, intravenous; PEA, pulseless electrical activity; ROSC, return of spontaneous circulation.

Median (IQR) EMS response time was 5 (4-7) minutes, time on scene was 14 (8-21) minutes, and transport time was 10 (6-15) minutes. Overall, 529 patients (52%) had an endotracheal tube placement attempt, and 149 patients (15%) had attempted supraglottic airway placement. Vascular access (intravenous [IV] or intraosseous [IO]) was successfully established in 854 patients (84%) overall, but only in 6 of 42 birth-related cases (14%). IO access was successfully established in 729 of 814 encounters (90%) during which it was attempted, while IV access was successful in 165 of 276 attempted encounters (60%). A total of 816 patients (80%) received epinephrine, 57 (6%) received dextrose, and 51 (5%) received sodium bicarbonate (eTable 1 in Supplement 1). Overall, 202 patients (20%) had sustained ROSC, 169 (17%) were declared deceased before arrival to a hospital, and 648 (64%) arrived at the hospital with ongoing resuscitation.

**Figure 2.**
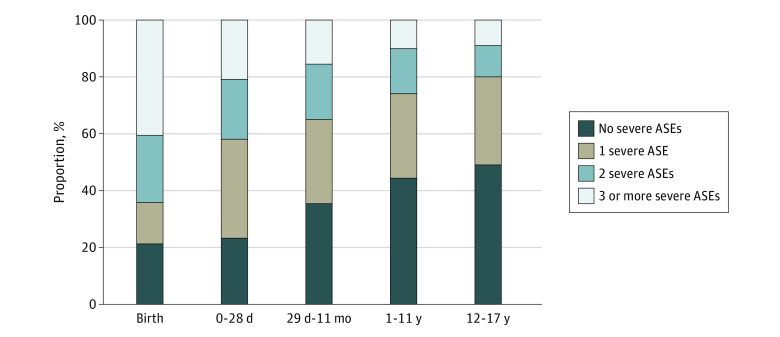
Percentage of Encounters With No, 1, 2, and 3 or More Severe Adverse Safety Events (ASEs) Among 1019 Children With Emergency Medical Services–Treated Out-of-Hospital Cardiac Arrest, by Patient Age

We identified a total of 1116 severe ASEs. Overall, 610 patients (60%) had at least 1 severe ASE, and 310 (30%) had 2 or more severe ASEs (Figure 2). While 98 of 200 adolescents (49%) with OHCA had no severe ASEs, only 19 of 43 neonates (22%) 28 days or younger (including birth-related and non–birth-related OHCA) had no severe ASEs. The most common severe ASE was delayed epinephrine administration (ie, not given within 10 minutes of EMS arrival). Overall, 332 encounters (30%) had either delayed administration of epinephrine or failure to give epinephrine even though it was indicated (Table 2; eTable 2 in Supplement 1). A total of 128 encounters (11%) had a delay in providing ventilation, while 125 (11%) had delayed vascular access.

**Table 2.** Most Frequent Severe ASEs Among 1116 Total Severe ASEs

Characteristic	Severe ASEs, No. (%) (n = 1116)
Severe ASEs in each domain of care	
Assessment	44 (4)
Clinical decision-making	22 (2)
Procedures	237 (21)
Airway	352 (32)
Medications	443 (40)
Fluids	16 (1)
Most frequently occurring severe ASEs	
Delay in giving epinephrine (>10 min)	244 (22)
Delay in ventilation (>2 min)	128 (11)
Delay in establishing vascular access (>10 min)	125 (11)
Epinephrine indicated and not given	89 (8)
Wrong dose of medication given (>10-fold overdose or underdose)	89 (8)
Unsuccessful advanced airway placement	53 (5)
Incorrect airway size	40 (4)
Failure to establish vascular access	39 (3)
Unsuccessful vascular access procedure	37 (3)
Failure to ventilate	32 (3)
Failure to adequately confirm advanced airway placement with capnography	27 (2)
Multiple attempts required to complete airway procedure (≥3 attempts)	25 (2)
Inappropriate use or failure to use specific American Heart Association protocol	17 (2)
Inappropriate monitoring of vital signs	12 (1)
Fluids indicated and not given	11 (1)
Multiple attempts required to establish vascular access (≥3 attempts)	11 (1)

Abbreviation: ASE, adverse safety event.

Among 80 encounters undergoing dual review, agreement between reviewers was 93% for assessment, 91% for clinical decision-making, 87% for nonairway procedures, 66% for airway procedures, 78% for medications, and 98% for fluids. Disagreements for airway procedures were primarily related to the assessment of whether ventilation of the patient was delayed.

In our logistic regression model quantifying the association between patient characteristics and severe ASEs clustered by study site (Table 3), neonates with birth-related OHCA had greater odds of a severe ASE compared with adolescents (OR, 7.0; 95% CI, 3.1-16.1). Neonates with non–birth-related OHCA also had greater odds of a severe ASE compared with adolescents (OR, 3.4; 95% CI, 1.2-9.6). We did not identify an association between other patient characteristics, OHCA characteristics, EMS response time or scene time, and severe ASEs.

**Table 3.** Factors Associated With 1 or More Severe Adverse Safety Events in Unadjusted Analysis and in Multivariable Model of 1019 Encounters of Pediatric Emergency Medical Services–Treated Out-of-Hospital Cardiac Arrest

Factor	No.^a^	OR (SE) [95% CI]	Adjusted OR (SE) [95% CI]
Patient age category			
Births	979	3.5 (1.4) [1.6-7.7]	7.0 (3.0) [3.1-16.1]
Non-birth neonates		3.2 (1.2) [1.5-6.8]	3.4 (1.8) [1.2-9.6]
Infants		1.7 (0.3) [1.2-2.5]	1.8 (0.6) [0.9-3.6]
Children		1.2 (0.2) [0.9-1.7]	1.3 (0.4) [0.7-2.3]
Adolescents		1 [Reference]	1 [Reference]
Male sex	1013	1.0 (0.1) [0.8-1.3]	1.0 (0.2) [0.7-1.4]
Witnessed arrest	957	1.0 (0.1) [0.7-1.2]	0.8 (0.1) [0.6-1.1]
Bystander CPR	1008	0.8 (0.1) [0.6-1.1]	0.9 (0.2) [0.6-1.3]
Time to scene, min	965	1.0 (0.02) [1.0-1.0]	1.0 (0.02) [1.0-1.1]
Time on scene, min	944	1.0 (0.003) [1.0-1.0]	1.0 (0.01) [1.0-1.0]
Public location	1019	1.1 (0.2) [0.8-1.4]	1.1 (0.2) [0.8-1.5]
Complex chronic condition	1019	1.0 (0.1) [0.7-1.3]	1.1 (0.1) [0.9-1.5]
Shockable rhythm	973	1.1 (0.3) [0.6-1.8]	1.3 (0.3) [0.9-2.1]

Abbreviations: CPR, cardiopulmonary resuscitation; OR, odds ratio.

^a^ Sample size for each analysis varies depending on No. of samples excluded due to missing data.

## Discussion

In our geographically diverse cohort of more than 1000 care episodes for US children with EMS-treated OHCA, 60% of all patients experienced at least 1 ASE that could cause permanent or severe harm during their EMS care. The most common severe ASEs were related to medication administration, airway procedures, and establishing vascular access.

This large study confirms findings from smaller studies that found a high rate of severe ASEs in pediatric OHCA. A prior study identified 1 or more severe ASEs in 21 of the 32 children (66%) with OHCA.^17^ Similar findings have been observed in studies of pediatric resuscitation by hospital personnel, with high rates of nonadherence to AHA guidelines.^18,19,20,21,22^ Potential contributors include the relatively low incidence of pediatric resuscitation and procedures, weight-based medication dosing, different equipment sizes and skills needed to successfully perform critical procedures, and unique stressors related to the care of critically ill children.^23^

Our large sample size allowed us to evaluate which severe ASEs were most prevalent in OHCAs. Medication errors represented 40% of all severe ASEs, in line with previous studies in prehospital and inpatient settings.^20,24,25,26^ The most common specific severe ASEs we identified involved administration of epinephrine, a key component of high-quality care for children with cardiac arrest, with several studies suggesting improved outcomes if epinephrine is given early in resuscitation.^27,28,29,30^ Successful administration of epinephrine to younger children in accordance with AHA guidance requires that EMS clinicians establish vascular access, then determine the correct size-based dose; failure to perform either of these effectively can result in epinephrine not given, delays, or 10-fold dosing errors. Interventions focused on earlier delivery of a correct dose of epinephrine may decrease these ASEs and improve outcomes. Several interventions, including cognitive aids, volume-based dosing guides, and prefilled syringes, have been suggested to improve accuracy and timing of medications administered to children.^31,32,33,34,35,36,37,38,39^ Our findings highlight the opportunity to focus quality improvement and educational interventions on early administration of epinephrine during pediatric OHCA.

We also identified many severe ASEs in vascular access and airway procedures. These procedures require appropriate size-based equipment, and EMS personnel may be less technically proficient in these procedures in younger children than adults. We observed that 32% of severe ASEs were airway management errors, with the largest number related to delays in providing positive pressure ventilation. Ventilation is relatively more important in pediatric patients compared with adults due to differing physiology and arrest etiology. It is possible that recent deemphasis on ventilation in adult OHCA has affected pediatric care, potentially causing harm. Importantly, delays in ventilation were often difficult to confirm using PCRs due to inconsistent documentation of these procedures, leading to lower interrater agreement in our study. We observed relatively fewer ASEs related to intubation than in our prior work, which could be due to the high rates of bag-mask ventilation only in this cohort.^9^

EMS teams may be more likely to establish IO than IV access in children. We found that success of IO catheter placement was 30% higher than IVs.^40^ A recent meta-analysis of adult OHCA studies identified no difference in hospital survival or neurologic outcome between IO and IV access strategies,^41^ but the increased difficulty of establishing IV in younger children may favor an initial IO approach for this population.

Our multivariable analyses identified neonates and newborn deliveries as having the highest odds of severe ASEs. This is concerning given that the number of births outside of the hospital is increasing. Recent data from the National Center for Health Statistics shows a 19% increase in home births nationally, from 38 506 in 2019 to 45 646 in 2020.^42^ Out-of-hospital births are associated with more than twice the risk of death and severe morbidity compared with in-hospital births.^43,44^ EMS personnel may be the primary and most experienced medical practitioners at these deliveries when the 911 system is activated, but they have limited exposure to birth emergencies. The resuscitation algorithm for births is markedly different than for other children and may not be widely disseminated among EMS personnel.^45^ In addition, planned home births during which 911 is activated represent situations where a complication has been identified by the delivery staff, potentially raising the risk of adverse outcomes. It is critical for EMS personnel responding to obstetric emergencies to be prepared to manage both the obstetric emergency and the resuscitation needs of the infant. Given the infrequency of events and cost of training, real-time decision support may be a promising strategy to improve performance.

### Limitations

There are limitations of this study. The structured and consistent design of data elements in EMS PCRs improves data quality and consistency. However, our findings are based on what is documented in the record. While EMS PCR fields adhere to national standards, agencies may have different documentation practices that could affect the likelihood of detecting some severe ASEs. Our decision to exclude encounters with insufficient documentation to determine whether a severe ASE occurred may have resulted in selection bias.^46^ In addition, differences in clinical protocols and local EMS practice may contribute to differences in the frequency of specific ASEs.

We discovered medical record deficiencies that raise the question of which data fields ought to be structured. While structured fields easily indicate medication names, doses, and routes, documentation of bag mask ventilation currently relies on unstructured reporting. This may have led to inconsistent reporting of bag mask ventilation, making it difficult to more completely and reliably assess whether this critical component of pediatric OHCA care was performed in a timely manner. Structured data fields increase the length of reports and can increase documentation burden. Thus, it is important to consider the value of those data and the likelihood that a structured field would improve accuracy. While structured data fields in electronic records can also serve as a prompt or reminder of care delivery, this may have more limited value in the prehospital setting given that most documentation by EMS clinicians occurs after the care episode.

Another limitation of our study is that EMS PCRs do not contain enough information to assess whether key elements of care (eg, chest compressions) were performed correctly. Additionally, as with any medical record review, our process relies on the best clinical judgment of experts. While clinical experts are credible judges of care delivery, judgment allows room for variation. We utilized several safeguards to minimize this risk: (1) our process was based on 2 of the most respected patient safety studies conducted in medicine^47,48^; (2) we developed structured data forms based on these studies, creating discrete variables for data collection (minimizing variation that could happen through narratives alone); (3) all reviewers underwent a rigorous training process and were provided a matrix of criteria for common ASEs; and (4) we had ongoing quality control consisting of dual review of some medical records. Despite these safeguards, we found disagreement between reviewers, especially in airway procedures and medications. We chose conservative cutoffs for severe ASEs (eg, delivery of first epinephrine dose within 10 minutes of arrival) to minimize the likelihood that imperfect medical record–keeping or reviewer disagreement surrounding a specific cutoff would lead to overidentification of severe ASEs. As a result, our findings may represent an underestimate of the true number of severe ASEs.

We excluded care episodes during which resuscitation was stopped before 10 minutes, as most of these patients had obvious signs of death on EMS arrival and others had resuscitation stopped quickly. It is difficult to determine from medical record review whether some patients with these limited resuscitations were potentially viable with ideal care. In addition, while all care episodes in this study were eligible for most ASEs, the total number of potential ASEs for each episode may differ based on factors, such as duration of the episode. Additionally, while our findings represent a broad geographic sample within the United States, they may not be applicable to other areas or countries due to differences in EMS response models, pediatric training, equipment, and other factors.

## Conclusions

In this large, geographically diverse cohort of children with EMS-treated OHCA, 60% experienced at least 1 severe ASE. These findings represent an urgent call to action to improve overall training and delivery of care, with special emphasis on neonates and birth-related cardiac arrests.

## Notes

Supplement 1. **eTable 1.** Medications Given During Resuscitation of 1019 Encounters of Pediatric EMS-Treated OHCA **eTable 2.** Most Frequent Severe ASEs by Age Group Among 1116 Total Severe ASEs

Supplement 2.
Nonauthor Collaborators

Supplement 3.
Data Sharing Statement
