# Supplementary material for: Adverse Safety Events in Emergency Medical Services Care of Children With Out-of-Hospital Cardiac Arrest
Source: JAMA Netw Open. 2024 Jan 12;7(1):e2351535. doi: 10.1001/jamanetworkopen.2023.51535 (PMC10787316; doi:10.1001/jamanetworkopen.2023.51535)
Supplement: Supplement 1. — eTable 1. Medications Given During Resuscitation of 1019 Encounters of Pediatric EMS-Treated OHCA eTable 2. Most Frequent Severe ASEs by Age Group Among 1116 Total Severe ASEs [file jamanetwopen-e2351535-s001.pdf]

Supplemental Online Content

Eriksson C, Bahr N, Meckler G, et al; Child Safety Initiative–Emergency Medical Services for Children. Adverse safety events in emergency medical services care of children with out-of-hospital cardiac arrest. *JAMA Netw Open.* 2024;7(1):e2351535. doi:10.1001/jamanetworkopen.2023.51535

eTable 1. Medications Given During Resuscitation of 1019 Encounters of Pediatric EMS-Treated OHCA

eTable 2. Most Frequent Severe ASEs by Age Group Among 1116 Total Severe ASEs

This supplemental material has been provided by the authors to give readers additional information about their work.

© 2024 Eriksson C et al. *JAMA Network Open.*

eTable 1. Medications Given During Resuscitation of 1019 Encounters of Pediatric EMS-Treated OHCA

	**Neonates**		**Infants** (29 d - 11 mo) (n=380)	**Children** (1-11 years) (n=354)	**Adolescents** (12-17 years) (n=200)	**Total** (n=1,019)
	**Births** (n=42, 4%)	**Non-birth** (<28 days) (n=43)				
Epinephrine	7 (17%)	34 (79%)	321 (84%)	286 (81%)	168 (84%)	816 (80%)
Dextrose		3 (6%)	23 (6%)	21 (5%)	10 (5%)	57 (6%)
Sodium bicarbonate		1 (2%)	12 (3%)	18 (5%)	20 (10%)	51 (5%)
Naloxone	1 (2%)		4 (1%)	10 (2%)	25 (13%)	40 (3%)
Midazolam				6 (1%)	13 (7%)	19 (1%)
Atropine			3 (0%)	7 (1%)	5 (3%)	15 (1%)
Vasopressin					10 (5%)	10 (0%)
Succinylcholine				4 (1%)	5 (3%)	9 (0%)
Lidocaine				2 (0.6%)	4 (2%)	6 (0%)
Fentanyl				1 (0.3%)	1 (0.5%)	2 (0%)
Other		2 (4%)	7 (1%)	25 (7%)	38 (19%)	72 (7%)

© 2024 Eriksson C et al. *JAMA Network Open.*

eTable 2. Most Frequent Severe ASEs by Age Group Among 1116 Total Severe ASEs

	**Neonates**		**Infants** (29 d - 11 mo) (n = 453)	**Children** (1-11 years) (n = 332)	**Adolescents** (12-17 years) (n = 171)	**Total** (%) (n=1,116)
	**Births** (n = 100)	**Non-birth** (n = 60)				
**Total number of severe ASEs in each domain of care**						
Assessment	3 (3%)	2 (3%)	19 (4%)	11 (3%)	9 (5%)	44 (4%)
Clinical decision-making	8 (8%)	1 (2%)	7 (2%)	5 (2%)	1 (1%)	22 (2%)
Procedures	27 (27%)	19 (32%)	87 (19%)	70 (21%)	34 (20%)	237 (21%)
Airway	26 (26%)	11 (18%)	145 (32%)	110 (33%)	60 (35%)	352 (32%)
Medications	36 (36%)	26 (43%)	193 (43%)	128 (39%)	62 (36%)	445 (40%)
Fluids	0 (0%)	1 (2%)	2 (0.4%)	8 (2%)	5 (3%)	16 (1%)
**Most frequently occurring severe ASEs**						
Delay in giving epinephrine (> 10 minutes)	9 (9%)	13 (22%)	102 (23%)	76 (23%)	44 (26%)	244 (22%)
Delay in ventilation (> 2 minutes)	8 (8%)	3 (5%)	53 (12%)	33 (10%)	31 (18%)	128 (11%)
Delay in establishing vascular access (> 10 minutes)	5 (5%)	8 (13%)	47 (10%)	44 (13%)	21 (12%)	125 (11%)
Epinephrine indicated and not given	20 (20%)	8 (13%)	30 (7%)	23 (7%)	8 (5%)	89 (8.0%)
Wrong dose of medication given (>10-fold overdose or underdose)	6 (6%)	4 (7%)	53 (12%)	22 (7%)	4 (2%)	89 (8.0%)
Unsuccessful advanced airway placement	2 (2%)	1 (2%)	31 (7%)	15 (5%)	4 (2%)	53 (4.7%)
Incorrect airway size	1 (1%)	3 (5%)	15 (3%)	18 (5%)	3 (2%)	40 (3.6%)
Failure to establish vascular access	15 (15%)	2 (3%)	12 (3%)	5 (2%)	5 (3%)	39 (3.4%)
Unsuccessful vascular access procedure	6 (6%)	5 (8%)	16 (4%)	10 (3%)	0 (0%)	37 (3.3%)
Failure to ventilate	5 (5%)	1 (2%)	11 (2%)	9 (3%)	6 (4%)	32 (2.9%)
Failure to adequately confirm advanced airway placement with capnography	0 (0%)	1 (2%)	13 (3%)	5 (2%)	8 (5%)	27 (2.4%)
Multiple attempts required to complete airway procedure (3 attempts or more)	4 (4%)	0 (0%)	9 (2%)	10 (3%)	2 (1%)	25 (2.2%)

© 2024 Eriksson C et al. *JAMA Network Open.*

Inappropriate use or failure to use specific American Heart Association protocol	8 (8%)	0 (0%)	5 (1%)	3 (1%)	0 (0%)	17 (1.5%)
Inappropriate monitoring of vital signs	2 (2%)	0 (0%)	2 (0.4%)	4 (1%)	4 (2%)	12 (1.1%)
Fluids indicated and not given	0 (0%)	1 (2%)	0 (0%)	5 (2%)	5 (3%)	11 (1.0%)
Multiple attempts required to establish vascular access (3 attempts or more)	0 (0%)	1 (2%)	3 (1%)	5 (2%)	2 (1%)	11 (1.0%)

© 2024 Eriksson C et al. *JAMA Network Open.*
