# Supplementary material for: Adverse Safety Events in Emergency Medical Services Care of Children With Out-of-Hospital Cardiac Arrest
Source: JAMA Netw Open. 2024 Jan 12;7(1):e2351535. doi: 10.1001/jamanetworkopen.2023.51535 (PMC10787316; doi:10.1001/jamanetworkopen.2023.51535)
Supplement: Supplement 2. — Nonauthor Collaborators [file jamanetwopen-e2351535-s002.pdf]

Supplemental Online Content: Nonauthor Collaborators
*First name, last name, and suffix (if applicable) are required and will appear in PubMed.

***Group Name(s): Child Safety Initiative-Emergency Medical Services for Children**

***First Name and Middle Initial(s)**	***Last Name**	***Suffix (eg, Jr, III)**	Academic Degrees	Institution	Location (city, state/province, country)	Role or Contribution, eg, chair, principal investigator	Group (if more than 1 Group listed in the byline) and/or Subgroup (eg, Steering Committee)
Tabria	Harrod		MPH	Maternal Child and Family Program, Washington County		Project Manager	Child Safety Initiative - Emergency Medical Services for Children
Amanda	Schoonover		MPH	Michigan State University		Research Assistant	Child Safety Initiative - Emergency Medical Services for Children
Jon	Ivankovic		BS	Oregon Health & Science University		Research Assistant	Child Safety Initiative - Emergency Medical Services for Children
