# Supplementary material for: Adverse Safety Events in Emergency Medical Services Care of Children With Out-of-Hospital Cardiac Arrest
Source: JAMA Netw Open. 2024 Jan 12;7(1):e2351535. doi: 10.1001/jamanetworkopen.2023.51535 (PMC10787316; doi:10.1001/jamanetworkopen.2023.51535)
Supplement: Supplement 3. — Data Sharing Statement [file jamanetwopen-e2351535-s003.pdf]

Data Sharing Statement

Eriksson. Adverse Safety Events in Emergency Medical Services Care of Children With Out-of-Hospital Cardiac Arrest. *JAMA Netw Open*. Published January 12, 2024.
doi:10.1001/jamanetworkopen.2023.51535

Data
Data available: No

Additional Information
Explanation for why data not available: Our data includes potentially identifiable information about both patients and EMS agencies.
